# Effects of residual black *wolfberry* fruit on growth performance, rumen fermentation parameters, microflora and economic benefits of fattening sheep

**DOI:** 10.3389/fvets.2024.1528126

**Published:** 2025-01-10

**Authors:** Liangzhong Hou, Pingping Duan, Yuxia Yang, Ali Mujtaba Shah, Jinlong Li, Congbin Xu, Tongjun Guo

**Affiliations:** ^1^Feed Research Institute of Xinjiang Academy of Animal Husbandry Sciences, Urumqi, China; ^2^College of Animal Science and Technology, Northwest A&F University, Xianyang, China

**Keywords:** residual black *wolfberry* fruit, *Duolang* sheep, growth performance, rumen microorganisms, economic benefits

## Abstract

**Introduction:**

The residual black wolfberry fruit (RBWF) is rich in nutrients and contains a diverse range of active substances, which may offer a viable alternative to antibiotics. This experiment was conducted to investigate the impact of varying levels of RBWF on the growth performance and rumen microorganisms of fattening sheep, and to quantify its economic benefits.

**Methods:**

In this experiment, 40 three-month-old and male Duolang sheep with an average weight of 29.85 kg, selected for their propensity to gain weight, were randomly assigned to one of four groups, with ten sheep in each group. To this end, each group was fed with a different proportion of RBWF (0%, 2%, 5%, 8%), and rumen fluid samples were collected to detect differences in fermentation parameters and microbial structure.

**Results:**

The findings indicate that the dry matter intake, OM and NDF apparent digestibility of Duolang sheep in the H2 group were found to be significantly higher than those observed in the other groups (P < 0.05). The concentration of volatile fatty acids (VFAs), including acetate, propionate, iso-butyrate, butyrate and iso-valerate, in rumen fluid demonstrated a linear increase with the supplementation of RBWF in the diet (P < 0.05). The dominant bacteria in the rumen of Duolang sheep were identified as Prevotella, Christensenellaceae R7 group, NK4A214 group, Ruminococcus, and Rikenellaceae RC9 gut group. Compared with the CK group, the relative abundance of Prevotella, NK4A214 group, unclassified Prevotellaceae and Lachnospiraceae NK3A20 group in the rumen of sheep in each experimental group increased to varying degrees. The gross profit of the H2 group was significantly higher than that of the other groups.

**Conclusion:**

In conclusion, the supplementation of RBWF has been demonstrated to enhance the growth performance of Duolang sheep, optimise rumen fermentation parameters, and ultimately increase gross profit, of which 5 % is the best.

## 1 Introduction

*Lycium barbarum* is a deciduous shrub belonging to the Solanaceae family (1). It is a widely utilized ingredient in the food and medical industries, due to the presence of bioactive metabolites in the branches, leaves and fruits (2). From a global perspective, China is the world’s foremost producer of *wolfberries*, accounting for over 80% of global cultivation. The majority of China’s *wolfberry* cultivation is concentrated in the provinces of Ningxia, Qinghai, Gansu and Xinjiang. It is reported that in 2023, the total area devoted to *wolfberry* cultivation in China will reach 1216.67 km^2^, with an estimated output of 1.4 Mt. of fresh fruit and 0.24 Mt. of dry fruit. In the production of fresh *wolfberry*, a residual quantity of 5 to 10% of the fruit is inevitable. This is typically discarded as waste, resulting in a considerable amount of wastage. The contents of crude protein (CP), crude fat (EE), crude ash (Ash), total carbohydrates and dietary fiber were found to be 9.2, 3.0, 5.6, 61.6 and 12.1%, respectively (3). Furthermore, *Lycium ruthenicum Murray* is a rich source of polyphenols, polysaccharides, alkaloids, anthocyanins and other biochemical components. These component confer a range of beneficial effects, including immune regulation, antioxidant activity, anti-aging, anti-tumor, anti-fatigue and anti-inflammatory actions (4). Zhao et al. (5) demonstrated that the inclusion of 0.6% *Lycium barbarum* polysaccharide (LBP) in the diet of dairy cows resulted in a notable enhancement in their production performance, accompanied by an augmentation in the body’s antioxidant capacity and immunity. Zhang et al. (6) discovered that fermented *wolfberry* residue not only enhances carcass weight in fattening sheep but also stimulates the expression of chemokines and immune-related pathways in sheep. Gan et al. (7) demonstrated that LBP can induce an immune response, thereby regulating the immune response to diseases such as cancer.

In comparison to red *wolfberry*, black *wolfberry* exhibited a higher concentration of phenols, concentrated tannins and monomeric anthocyanins, and also demonstrated enhanced anti-inflammatory and antioxidant activities (8, 9). Currently, there is a plethora of research exploring the utilization of by-products derived from red fruit *wolfberry*. However, despite the unique functional components present in black fruit *wolfberry*, it has not yet been subjected to the same degree of investigation. A substantial body of evidence attests to the potential value of incorporating red *wolfberry* by-products into livestock and poultry production (10). It may therefore be hypothesized that the inclusion of black *wolfberry* fruit in the diet of animals could have a beneficial effect on their production performance. The objective of this experiment was to investigate the impact of varying levels of residual black *wolfberry* fruit (RBWF) on the growth performance, nutrient apparent digestibility, rumen fermentation parameters, microflora and gross profit of *Duolang* sheep. This was done in order to establish a theoretical basis for the application of residual black *wolfberry* fruit in sheep production.

## 2 Materials and methods

### 2.1 Ethics committee approval

The study was carried out in accordance with the procedures sanctioned for this research, which have been approved by the Science and Technology Ethics Committee of Xinjiang Academy of Animal Sciences, China (ethics number 20230508). These procedures adhere to the principles and regulations for ethical protection in human and animal biological science and technology in China.

### 2.2 Experimental animals and group design

In this experiment, following the collection of the black *wolfberry* fruit in October 2022, the fruit that did not meet the quality standard was identified as RBWF. The nutritional components of RBWF were then determined, as illustrated in Table 1. Based on the principle of equal energy and nitrogen, four diets containing different proportions (0, 2, 5, and 8%) of RBWF were formulated (NRC 2007).

**Table 1:** The main nutritional components of RBWF (DM basis, %).

Items	CP	EE	Ash	CF	ADF	NDF	Ca	Pi
RBWF	14.29	11.46	9.20	8.86	16.60	28.00	0.24	0.30

Forty male lambs, 3 months aged and with a mean body weight of 29.85 ± 2.00 kg, were used in this experiment. Following deworming, a single-factor completely randomized experimental design was implemented, with the lambs randomly divided into four groups, each with 10 replicates. Four distinct proportions of RBWF diets were provided, comprising the following: a control group (CK, 0% RBWF), experimental group 1 (H1, 2% LBL), experimental group 2 (H2, 5% LBL) and experimental group 3 (H3, 8%LBL). The specific diet composition and nutrient composition are presented in Table 2. The experiment spanned 70 days, comprising a 10-day pre-experimental period and a 60-day experimental period.

**Table 2:** Composition and nutrient levels of the basal diet (DM basis, %).

Items	CK	H1	H2	H3
Ingredients				
Corn	33.80	35.00	32.50	31.00
Wheat bran	9.00	6.40	6.28	5.15
cottonseed meal	13.70	13.30	12.92	12.65
RBWF	0.00	2.00	5.00	8.00
Corn stalk	18.00	18.00	18.00	17.90
Alfalfa	20.50	20.30	20.30	20.30
Premix^1^	5.00	5.00	5.00	5.00
Total	100.00	100.00	100.00	100.00
Nutritional level^2^				
ME (MJ/kg)	11.06	11.02	10.98	10.93
CP	13.96	13.97	13.99	13.99
NDF	31.12	31.14	31.24	31.45
ADF	16.80	16.85	17.07	17.30
Ca	0.70	0.93	1.16	1.10
P	0.49	0.56	0.64	0.41

1 The premix provided the following per kg of the diet: VA 150,000 IU, VD3 56,500 IU, VE 8,000 IU, Se (as sodium selenite) 14 mg, I (as potassium iodide) 58 mg, Cu (as copper sulfate) 290 mg, Mn (as manganese sulfate) 1,925 mg, Zn (as zinc oxide) 2,050 mg, Co (as cobalt sulfate) 24 mg.

2 Nutritional level were measured values.

### 2.3 Feeding management

Prior to the commencement of the experiment, the sheep house was thoroughly cleaned and disinfected, and the test sheep were subjected to a series of preparatory procedures, including cutting, deworming, and medicated bathing. During the course of the experiment, the sheep were maintained in separate columns and provided with food twice daily, at 10:00 and 18:00, respectively. The *Duolang* sheep were permitted to feed and drink freely throughout the duration of the experiment.

### 2.4 Sample collection and measurements

#### 2.4.1 Growth performance and apparent digestibility

The quantity of feed provided and the quantity of residual feed were recorded on a daily basis. Prior to the morning feeding on the 1st, 30th and 60th days of the experiment, each sheep was weighed. Thereafter, the average daily feed intake (ADFI), average daily gain (ADG) and feed-to-weight ratio (F:G) were calculated. A digestion test was conducted over the final 10 days of the formal test period, comprising a 5-day adaptation period. Fecal samples were collected using the total fecal collection method over 5 consecutive days. The total fecal matter of each sheep was weighed prior to morning feeding on a daily basis, with 10% of the total fecal matter collected from each sheep. Following the conclusion of the experiment, the collected fecal samples were combined in a uniform manner, with 10% of the samples obtained through the quartering method and stored at −20°C for subsequent analysis.

An analysis was conducted on samples of diet, ingredients, and feces for dry matter (method 930.15), CP (method 990.03), EE (method 920.39), Ca (method 978.02), and P (method 946.06) using the AOAC procedures (11). NDF and ADF content were determined following Van Soest’s (12) method. ME was calculated based on the measured nutritional value:


$$\mathrm{ME}=0.046+0.820\times \left(17.211-0.135\times NDF\right)$$

#### 2.4.2 Rumen fermentation parameters and microorganisms

Immediately following the formal test, the test sheep were slaughtered after 16 h of fasting and rumen fluid was collected. The pH value was immediately measured following filtration through four layers of gauze using a portable pH meter (PHS-3C, Shanghai, China), and the measurement was repeated 3 times. To assess rumen fermentation parameters and rumen microflora, the remaining samples were transferred to 15 mL and 5 mL freezing tubes and stored in −20°C and − 80°C freezers, respectively. The concentration of NH_3_-N was determined by phenol sodium hypochlorite colorimetry; the concentration of volatile fatty acids (VFAs) was determined by gas chromatography.

#### 2.4.3 Extraction of DNA and sequencing of 16S rDNA

The TGuide S96 Magnetic Bead Method Soil/Fecal Genomic DNA Extraction Kit (Tiangen, Beijing, China) was used to extract DNA from the rumen specimens for 16S rDNA sequencing analysis. The concentration of the extracted nucleic acids was determined using an enzyme marker (GeneCompany Limited, Hong Kong, China, model Synergy HTX), and their integrity was assessed through agarose electrophoresis at a concentration of 1.8% (Beijing Bomei Fuxin Technology Co., Ltd., Beijing, China).

The highly variable V3-V4 regions of bacterial 16S rDNA were amplified by PCR using universal bacterial primers 338F (5′- ACTCCTACGGGGAGGCAGCA-3′) and 806R (5′- GGACTACHVGGGGTWTCTAAT-3′) after extracting total DNA from the samples. The final products were cleaned, quantified, and mixed to form a sequencing collection. After passing quality assessments, the collection was analyzed on the Illumina NovaSeq 6,000 instrument (San Diego, CA, USA). Raw data files from various high-throughput sequencers, including the Illumina NovaSeq, were processed to generate sequenced reads. These reads include both the actual sequences and quality metrics. The initial reads were refined with the Trimmomatic v0.33 tool. To further refine the data, Cutadapt 1.9.1 was utilized to eliminate primer sequences and produce polished reads.

### 2.5 Statistical analysis

The preliminary sorting of the experimental data was conducted using Excel 2023 software, while the significance test was performed with the one-way ANOVA program in SPSS 26.0 statistical software. Subsequently, linear and quadratic analyses were carried out on rumen fermentation parameters, employing the Duncan method for multiple comparison differences. The level of significance was determined by *p* < 0.05, while 0.05 < *p* ≤ 0.10 indicated a trend. The taxonomy annotation of the OTUs was conducted by classifying representative organisms using a Bayesian classifier based on the SILVA database (version 138). Alpha diversity analyzes species diversity and complexity using ACE, Chao1, Simpson, and Shannon indices. Beta diversity analyses were conducted using principal coordinate analysis (PCoA) and nonmetric multidimensional scaling (NMDS) to evaluate distinctions between groups. LEfSe (Line Discriminant Analysis (LDA) Effect Size) was employed to facilitate a comparative analysis of the various treatment groups. The differences between the classification levels were then analyzed individually, from the lowest level (species) to the highest (phylum), with the objective of identifying biomarkers exhibiting statistically significant differences between the groups.

## 3 Results

### 3.1 Effects of varying levels of RBWF addition on the growth performance and nutrient apparent digestibility of *Duolang* sheep

Table 3 illustrates the impact of incorporating varying levels of RBWF into the diet on the growth performance of *Duolang* sheep. It can be observed that following a 60-day feeding period, the final weight gain of sheep in the H2 group was 14.89 kg, representing a 4.78, 12.97, and 13.32% increase compared to the CK, H1, and H3 groups (14.21 kg, 13.18 kg, and 13.14 kg, respectively). The H2 group exhibited the most favorable outcome, although no statistically significant difference was observed (*p* > 0.05). Concurrently, the dry matter intake of the H2 group was markedly higher than that of the CK group (*p* < 0.05).

**Table 3:** Effects of RBWF on growth performance of *Duolang* sheep (*n* = 10).

Items^1^	CK	H1	H2	H3	SEM	*p*-value^2^		
						Trt	L	Q
Initial body weights, kg	29.91	30.05	29.90	29.54	0.230	0.901	0.586	0.605
Final weight, kg	43.77	43.09	45.06	42.70	0.557	0.522	0.821	0.526
ADG, g	249.29	231.19	261.18	230.48	8.394	0.563	0.733	0.788
total gain weight, kg	14.21	13.18	14.89	13.14	0.478	0.563	0.733	0.788
ADFI, kg	1.79^b^	1.73^b^	1.84^a^	1.65^c^	0.043	0.021	<0.001	<0.001
F:G	7.52	7.78	7.23	7.67	0.271	0.925	0.976	0.909

1 ADG, average daily gain; ADFI, average daily feed intake; F:G, ADFI/ADG.

2 Trt, treatment effect; L, linear; Q, quadratic.

^a,b,c^ Different letters indicate significant differences between different groups (*p* < 0.05). SEM is the pooled standard error between five groups; the *p*-value indicates significance.

Table 4 demonstrated that dietary RBWF supplementation had no statistically significant impact on the apparent digestibility of dry matter (DM), crude protein (CP), ether extract (EE), acid detergent fiber (ADF), calcium (Ca) and phosphorus (P) in *Duolang* sheep when compared with the CK group (*p* > 0.05). However, the apparent digestibility of organic matter (OM) and NDF in the H2 group was found to be significantly higher than that observed in the H3 group (*p* < 0.05).

**Table 4:** Effect of RBWF on apparent digestibility of nutrients in *Duolang* sheep (*n* = 10).

Items	CK	H1	H2	H3	SEM	*p*-value^1^		
						Trt	L	Q
DM	70.60	68.80	71.20	66.90	0.638	0.059	0.099	0.277
OM	59.76^b^	58.64^b^	61.61^a^	56.46^c^	0.577	0.047	0.091	0.144
CP	73.26	73.04	73.45	72.63	0.173	0.397	0.342	0.396
EE	55.67	56.24	56.43	56.57	0.418	0.896	0.478	0.809
NDF	43.72^b^	44.76^ab^	47.55^a^	41.81^b^	0.677	0.010	0.536	0.005
ADF	24.93	25.88	27.74	23.69	0.634	0.135	0.728	0.048

1 Trt, treatment effect; L, linear; Q, quadratic.

^a,b,c^ Different superscripts indicate significant differences within a row (*p* < 0.05). SEM is the pooled standard error between five groups; the *p*-value indicates significance.

### 3.2 Effects of different levels of RBWF supplementation on rumen fermentation parameters in *Duolang* sheep

Table 5 illustrates the impact of dietary supplementation with RBWF on rumen fermentation parameters in *Duolang* sheep. It can be observed that no statistically significant difference was evident in pH and NH_3_-N concentration between the experimental groups (*p* > 0.05). In comparison to the CK group, the pH of the rumen in sheep exhibited a linear decline (*p* = 0.080), while the NH_3_-N concentration demonstrated a notable linear increase (*p* < 0.05). The concentrations of acetate and propionate in the rumen of the H2 group were found to be significantly higher than those observed in the other experimental groups. The concentrations of iso-butyrate, butyrate, iso-valerate and total volatile fatty acids (TVFAs) in the rumen of the H2 and H3 groups were significantly higher than those of the CK group (*p* < 0.05). As the ratio of RBWF supplementation increased, the concentrations of acetate, propionate, iso-butyrate, butyrate, iso-valerate, n-valeric acid and total volatile fatty acids (VFAs) in the rumen exhibited a linear increase (*p* < 0.05). However, the quadratic effect of acetate and iso-valerate was statistically significant (*p* < 0.05).

**Table 5:** Effects of RBWF on rumen fermentation parameters in *Duolang* sheep (*n* = 5).

Items^1^	CK	H1	H2	H3	SEM	*p*-value^2^		
						Trt	L	Q
pH	5.72	5.50	5.42	5.27	0.078	0.288	0.080	0.392
NH_3_-N, mg/100 mL	26.13	27.3	29.62	31.89	0.847	0.081	0.001	0.030
Acetate, mmol/L	88.49^c^	100.81^b^	115.63^a^	109.56^b^	3.964	<0.001	0.026	0.047
Propionate, mmol/L	27.34^c^	30.67^b^	33.54^a^	31.82^b^	1.104	<0.001	0.044	0.248
Iso-butyrate, mmol/L	0.87^c^	1.09^b^	1.43^a^	1.49^a^	0.082	0.002	0.045	0.272
Butyrate, mmol/L	20.43^c^	25.78^b^	31.59^a^	29.29^a^	1.608	0.005	0.046	0.064
Iso-valerate, mmol/L	1.34^c^	2.01^b^	2.83^a^	2.57^a^	0.186	0.001	0.035	0.032
Valerate, mmol/L	1.61	1.74	1.82	1.93	0.141	0.612	0.014	0.130
A:P	3.24	3.29	3.44	3.42	0.067	0.866	0.096	0.278
TVFAs, mmol/L	140.08^d^	162.10^c^	183.29^a^	176.92^ab^	6.485	<0.001	0.030	0.116

1 NH_3_-N, ammonia nitrogen; TVFA, total volatile fatty acids.

2 Trt, treatment effect; L, linear; Q, quadratic.

^a, b, c, d^ Different superscripts indicate significant differences within a row (*p* < 0.05). SEM is the pooled standard error between five groups; the *p*-value indicates significance.

### 3.3 Effects of different levels of RBWF supplementation on ruminal microbiota diversity analysis in *Duolang* sheep

**Figure 1:**
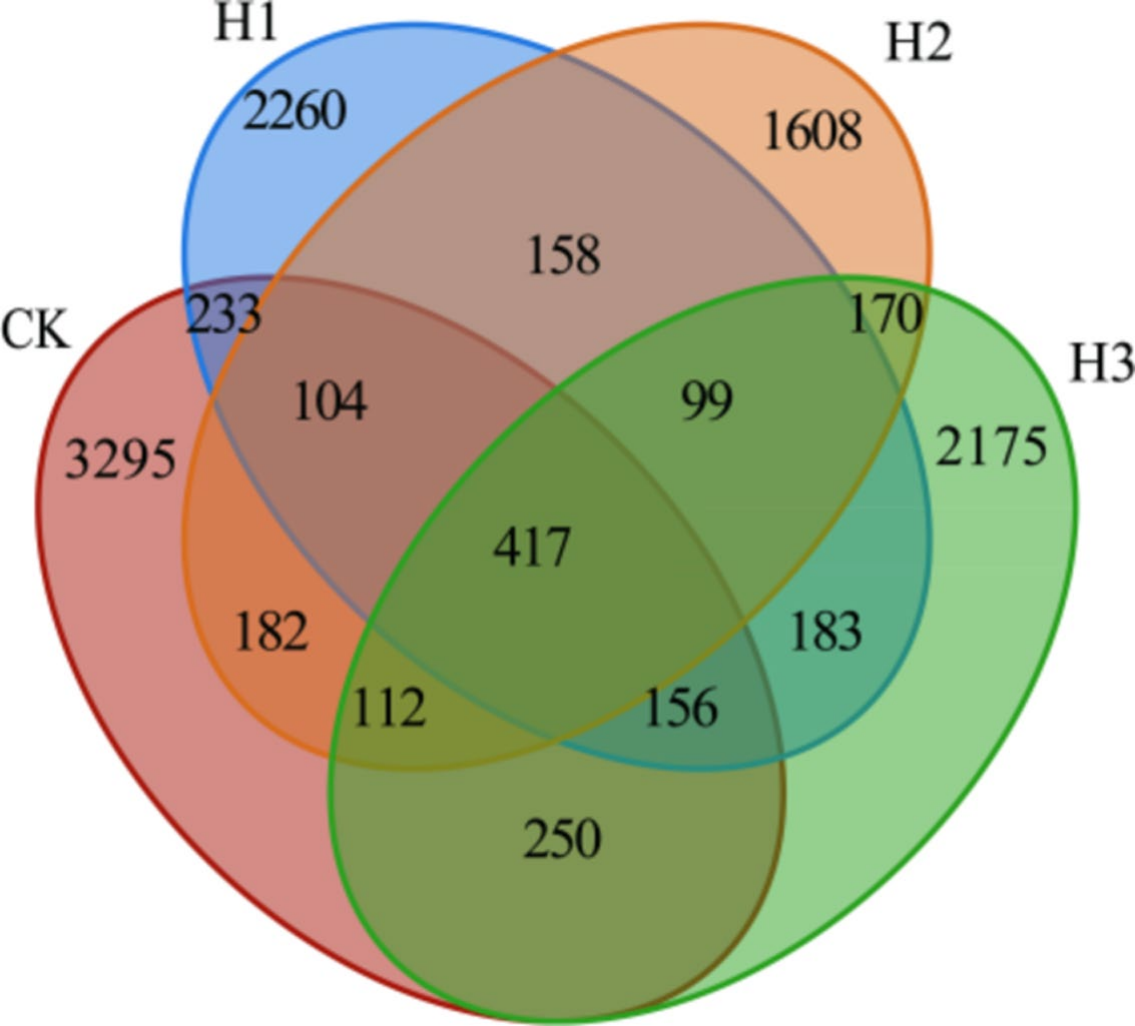
Venn diagram of ruminal fluid flora.

A total of 1,348,210 readings were obtained from 16 samples in four groups (5 in CK, 4 in H1, 3 in H2 and 4 in H3). Following quality control and splicing, 1,269,853 clean readings were obtained, with an average of 79,365 clean readings obtained for each sample. The number of unique OUTs in each group was as follows: 3,295 in CK, 2,260 in H1, 1,608 in H2 and 2,175 in H3. The total number of OUTs across all four groups was 4,170 (Figure 1).

**Figure 2:**
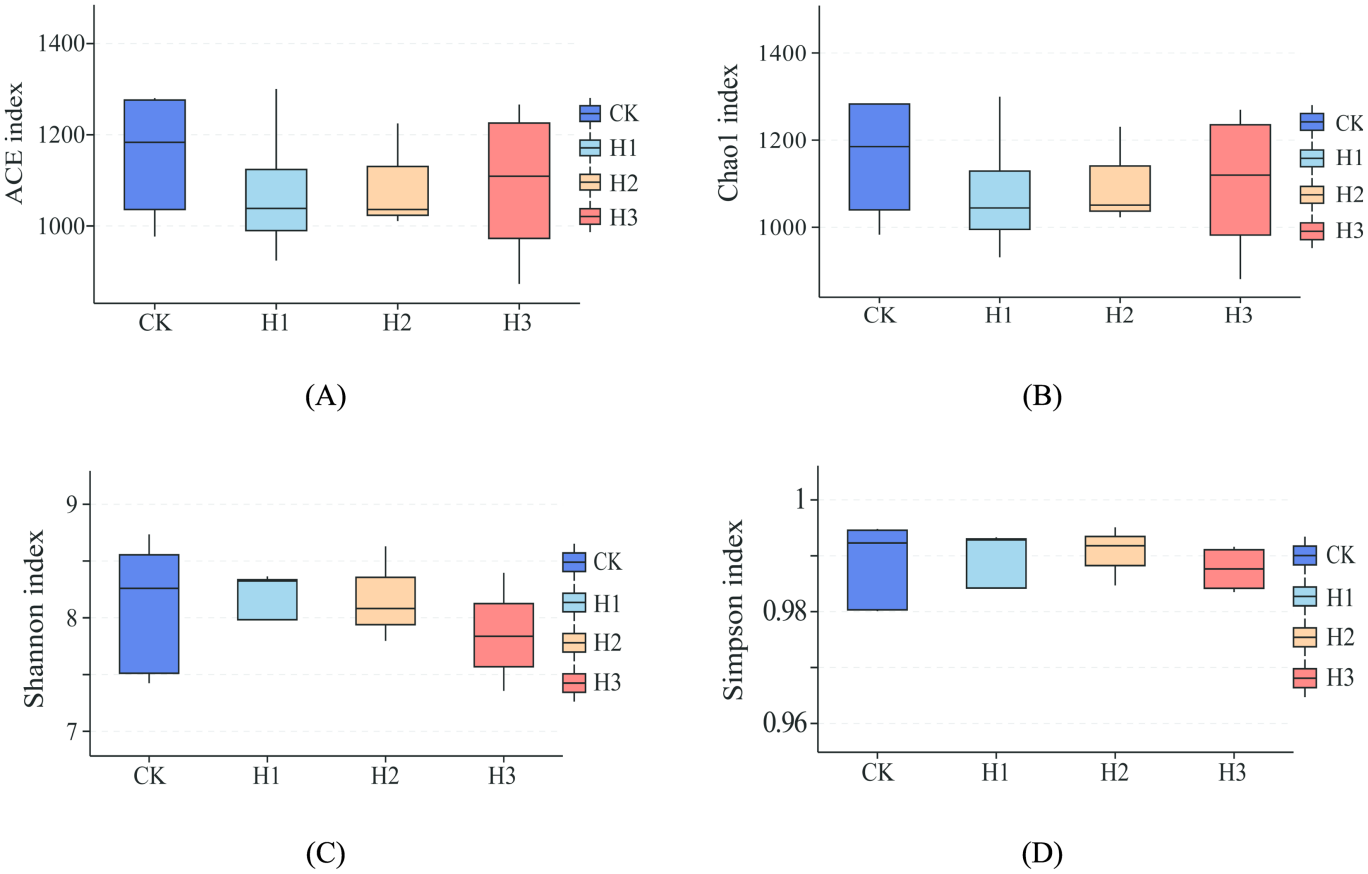
Alpha diversity analysis of rumen flora. **(A)** ACE index of species richness; **(B)** Chao1 index of species richness; **(C)** Shannon index of species diversity; **(D)** Simpson index of species diversity.

**Figure 3:**
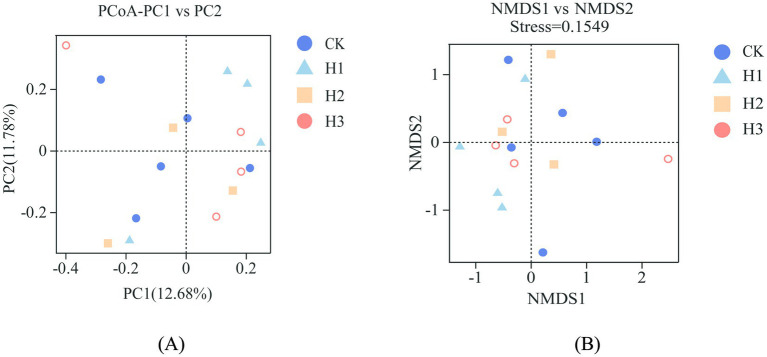
Beta diversity analysis of rumen flora. **(A)** PCoA principal axis analysis; **(B)** NMDS non-metric multidimensional scaling analysis.

As illustrated in Figure 2, dietary RBWF supplementation had no discernible impact on the alpha diversity of rumen microbiota in sheep. With regard to beta diversity, the results of PCoA (Figure 3A) and NMDS (Figure 3B) analysis demonstrated that there was no notable separation between the distribution of rumen microbial representative points across different treatment groups, thereby substantiating that the addition of RBWF had no substantial influence on the beta diversity of rumen microorganisms in *Duolang* sheep.

### 3.4 Effects of different levels of RBWF supplementation on analysis of microbial composition and community structure in *Duolang* sheep

**Figure 4:**
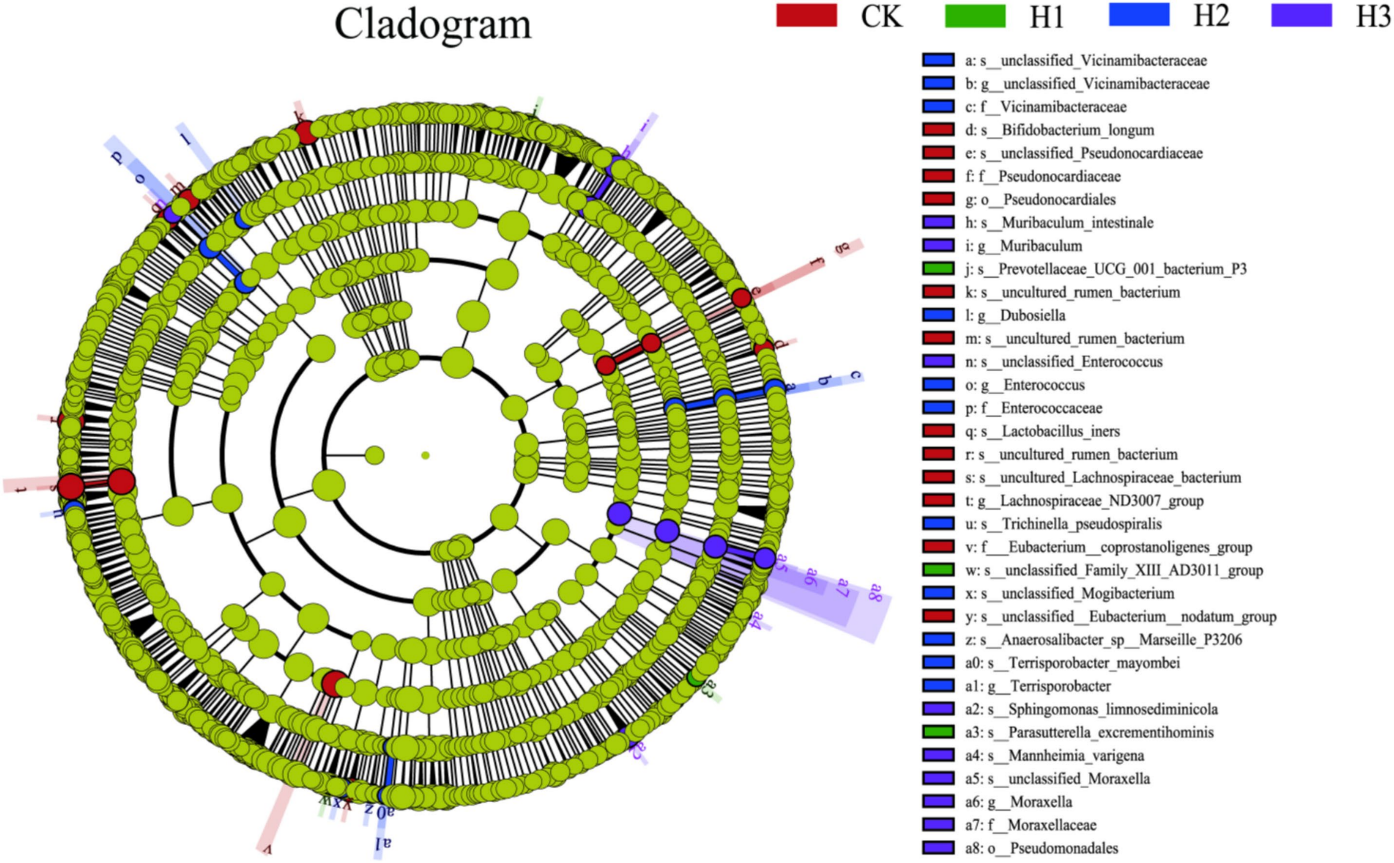
LEfSe (linear discriminant analysis effect size) cladogram comparing microbial communities among the 2.5 elevations.

**Figure 5:**
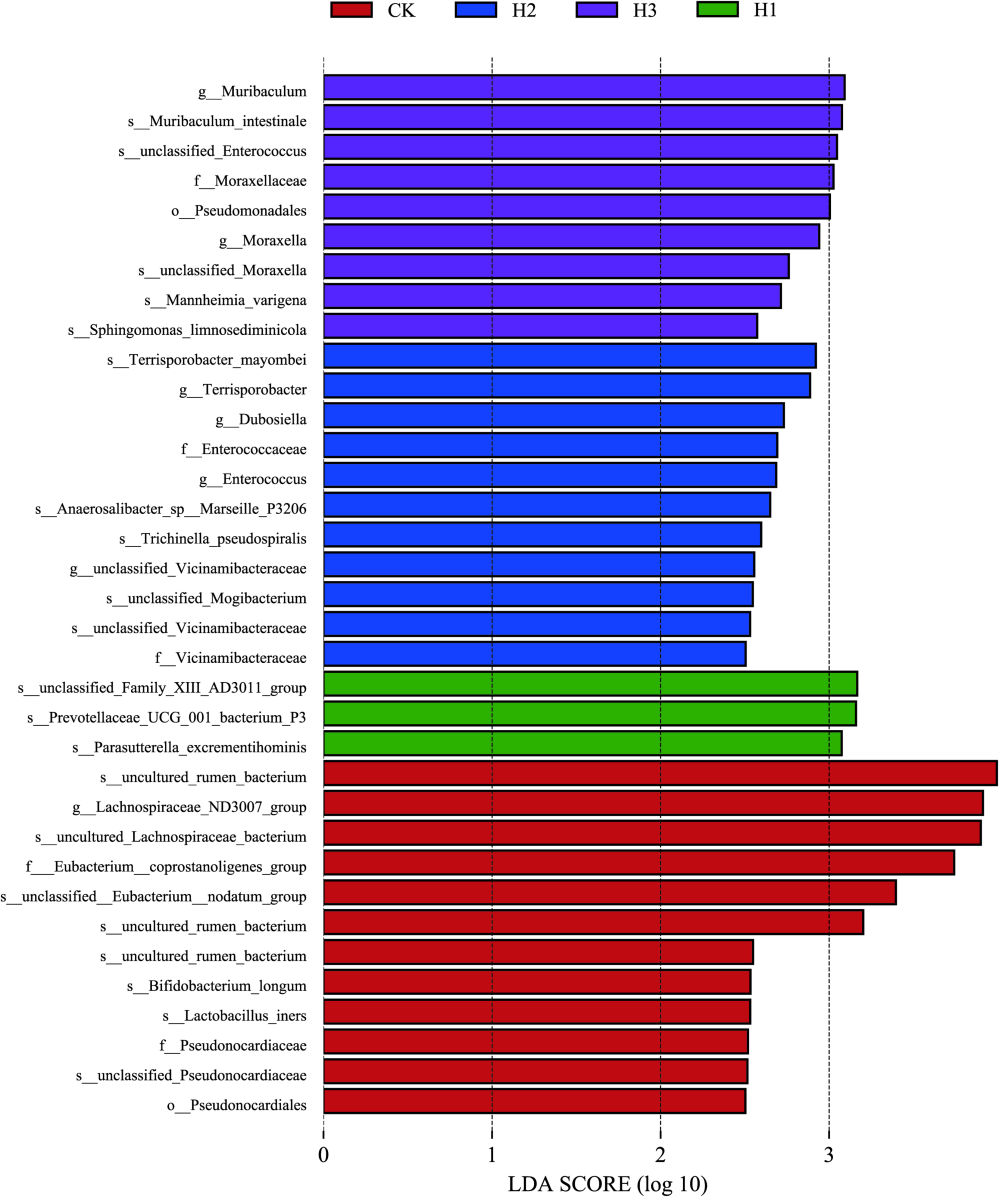
Histogram of LDA score calculated for each taxon ranging from phylum to genus.

A LEfSe (linear discriminant analysis effect size) analysis was conducted to identify discrepancies in the taxonomic composition of bacterial species. The figure presents a representative branch diagram of the main microbiome structure (Figure 4), illustrating the most notable differences between the groups of different treatment components at the phylum, class, order, family, genus, and species levels. The data indicated that 12 branches were more abundant in the CK group, 3 branches were more abundant in the H1 group, 11 branches were more abundant in the H2 group, and 9 branches were more abundant in the H3 group. The abundance differences of different bacterial groups among CK, H1, H2, and H3 are shown in Figure 5. The figure illustrates the abundance differences of various bacterial groups between the CK, H1, H2, and H3 groups. Notably, the *Lachnospiraceae ND3007 group*, *Eubacterium coprostanoligenes group*, and *Bifidobacterium longum exhibited* the most pronounced differences in abundance within the CK group. In contrast, the *Prevotellaceae UCG-001* and *Parasutterella excrementihominis genera* demonstrated the most significant abundance differences within the HC group. The bacterial genera *Terrisporobacter*, *Dubosiella* and *Enterococcus* were more abundant in H2, while *Muribaculum* and *Pseudomonadales* were more abundant in H3.

**Figure 6:**
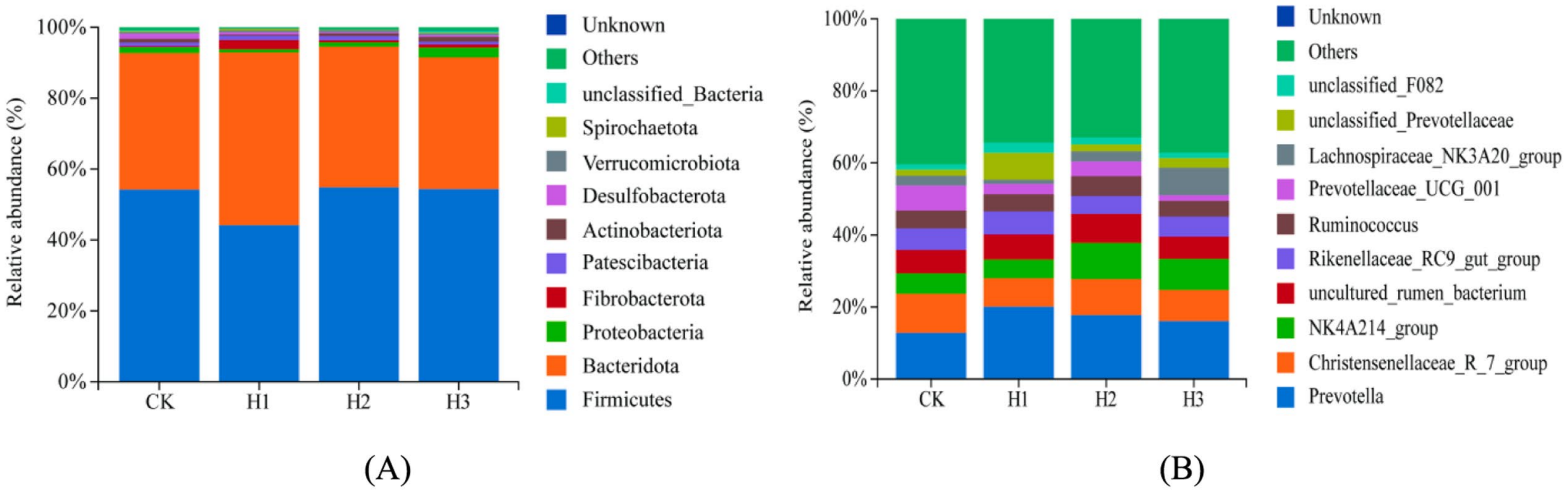
Distribution of bacterial taxa averaged under phyla **(A)** and genera **(B)** levels across the different treatment groups (as a percentage of the total sequence).

The differences in rumen microbial species abundance between the various treatment groups were subjected to analysis. At the phylum level (Figure 6A), the dominant bacterial species in the rumen of *Duolang* sheep were identified as *Firmicutes*, *Bacteroidota*, *Proteobacteria*, *Fibrobacterota*, and *Patescibacteria*. Following the addition of RBWF, no significant overall effect on the abundance of phylum-level flora was observed (*p* > 0.05). However, the relative abundance of *Bacteroidota* and *Fibrobacterota* in H1 group increased (Supplementary Table S1). At the genus level (Figure 6B), the dominant bacteria in the rumen bacteria of *Duolang* sheep were *Prevotella*, *Christensenellaceae R7 group*, *NK4A214 group*, *Ruminococcus*, and *Rikenellaceae RC9 gut group*. After the addition of RBWF, the overall effect on the abundance of genus-level bacteria was not significant (*p* > 0.05). Nevertheless, there was a notable increase in the relative abundance of *Prevotella*, *NK4A214 group* and *Lachnospiraceae NK3A20 group*, while the relative abundance of *Prevotellaceae UCG 001* decreased (Supplementary Table S2).

### 3.5 Correlation analysis of growth performance, nutrient apparent digestibility and rumen fermentation parameters with main bacteria at genus level

**Figure 7:**
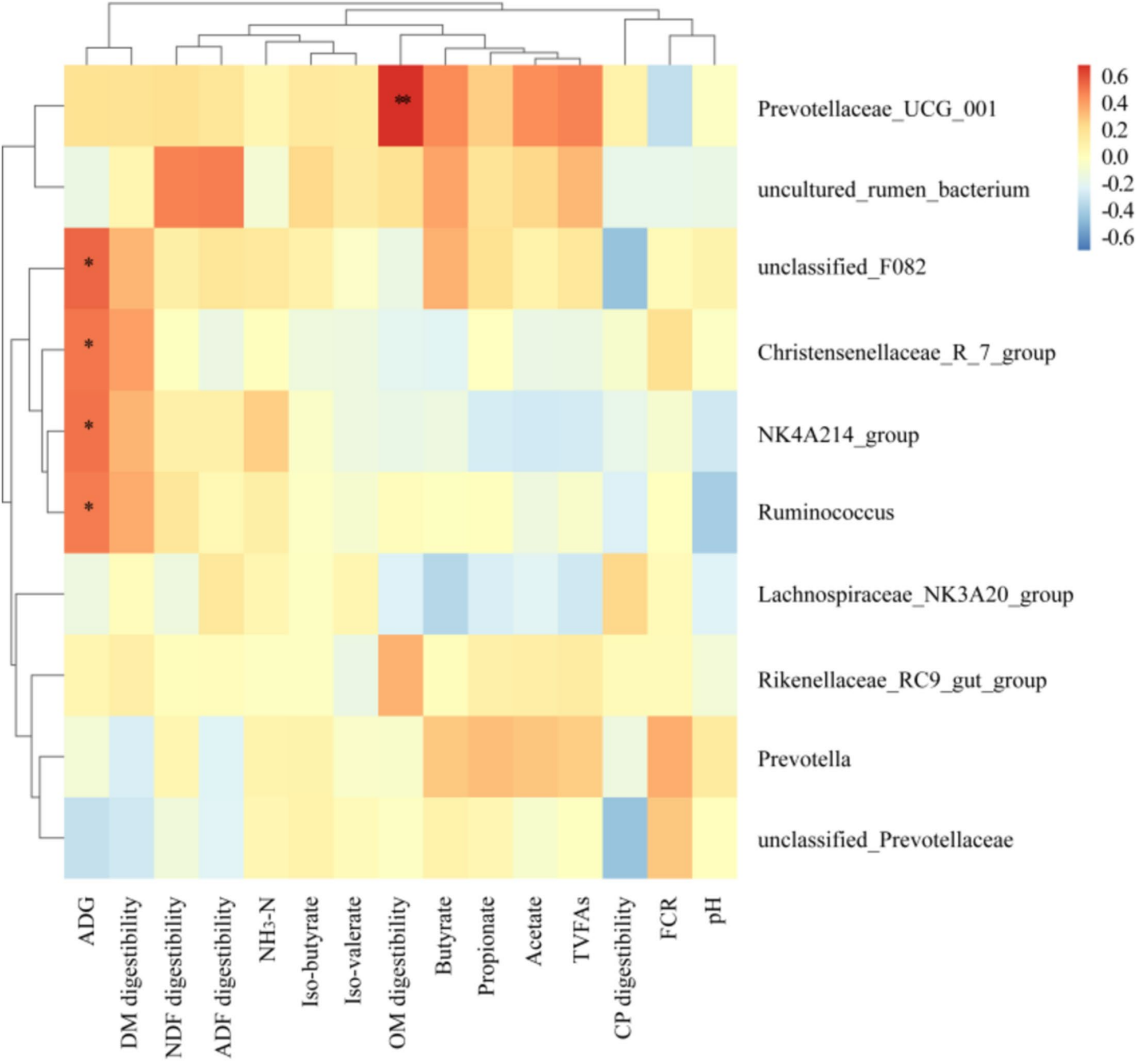
Spearman correlation and clustering analysis between growth performance, nutrient apparent digestibility and rumen fermentation parameters with main bacteria in Genus level. Different colors represent positive correlation (red) or negative correlation (blue), and the color shade indicates the magnitude of the correlation, * represents 0.01 < *p* ≤ 0.05 and ** represents *p* ≤ 0.01.

As illustrated in the Figure 7, the relative abundance of rumen-dominant bacteria exhibited a correlation with growth performance, nutrient apparent digestibility, and rumen fermentation parameters. The abundances of *F082*, *Christensenellaceae R7 group*, *NK4A214 group* and *Ruminococcus* were found to be significantly and positively correlated with ADG (*p* < 0.05). In contrast, *Prevotellaceae UCG 001* was observed to be negatively correlated with FCR and pH, and positively correlated with other indexes. Of these, it was found to be significantly and positively correlated with OM apparent digestibility (*p* < 0.05).

### 3.6 Effects of different levels of RBWF addition on economic benefits of *Duolang* sheep

The feed unit price of each group was found to be between 2.25 and 2.39 yuan/kg. As illustrated in the Table 5, as increase in the proportion of RBWF added to the diet was associated with a gradual decrease in feed unit price. The gross profit of the H2 group was observed to have increased by 19.34% in comparison to the CK group (Table 6).

**Table 6:** Effects of different levels of RBWF addition on economic benefits of *Duolang* sheep.

Items	CK	H1	H2	H3	SEM	*p*-value^1^		
						Trt	L	Q
Feed Unit price	2.39^a^	2.36^b^	2.30^c^	2.25^d^	0.009	<0.001	<0.001	<0.001
ADFI	1.79^b^	1.73^b^	1.84^a^	1.65^c^	0.043	0.021	<0.001	<0.001
Feed costs	257.12^a^	246.14^c^	242.75^b^	222.32^d^	2.238	<0.001	<0.001	<0.001
Sheep live weight unit price	31.00	31.00	31.00	31.00	0.000	-	-	-
Total gain weight, kg	14.21	13.18	14.89	13.14	0.478	0.563	0.733	0.788
Gain from weight gain	440.51	408.58	461.59	407.34	1.218	0.563	0.733	0.788
Net profit	183.39	162.46	218.86	185.02	1.979	0.789	0.743	0.968

1 Trt, treatment effect; L, linear; Q, quadratic.

^a, b,c,d^ Different letters indicate significant differences between different groups (*p* < 0.05). SEM is the pooled standard error between five groups; the *p*-value indicates significance.

- The unit price for live sheep weight is a fixed value that does not fluctuate.

## 4 Discussion

The current global concern surrounding antibiotic resistance is well documented (13). The intensification of animal husbandry practices at home and abroad has resulted in the pervasive use of antibiotics in animal husbandry production, which has introduced significant risks to food safety and environmental safety. The utilization of antibiotics in human and livestock production will inevitably result in the emergence of drug resistance (14). In order to mitigate the adverse effects of antibiotic resistance on human health, a number of healthcare institutions have introduced a series of policies aimed at promoting the rational use of antibiotics (15). *Lycium barbarum*, a traditional Chinese herbal medicine and health food, has been the subject of considerable interest due to the diversity of its functional components (16). Yin et al. (17) demonstrated that the inclusion of LBP in the diet of weaned piglets resulted in enhanced growth performance, antioxidant capacity, and immunity, as well as the regulation of intestinal microbial composition. Furthermore, they established that LBP can serve as an effective substitute for antibiotics in the feed of weaned piglets. Tian et al. (18) observed that *Lycium barbarum* partially alleviated the intestinal ecological imbalance caused by antibiotics by regulating the intestinal flora, and also produced short-chain fatty acids (SCFAs) to improve intestinal barrier function. Prior research has demonstrated that the bioactive components present in *Lycium barbarum* exhibit a range of biological functions that can prevent and treat chronic diseases, thereby positioning it as a potential alternative to antibiotics (19) The current research mainly focuses on the role of mining and extracting some active ingredients in *Lycium barbarum* as feed additives in livestock and poultry production and clinical practice (20, 21). However, the extraction method also affects the chemical properties of the active ingredients of *Lycium barbarum* (22). At the same time, there are few studies on the effects of *wolfberry* fruit on growth performance, nutrient apparent digestibility, rumen fermentation parameters and microbial flora structure of fattening sheep. Therefore, the objective of this study was to elucidate the impact of incorporating RBWF into the diet of fattening *Duolang* sheep and evaluate its economic benefits.

Prior research has demonstrated a significant correlation between the average daily feed intake of fattening sheep and feed conversion efficiency, with feed conversion efficiency being closely associated with growth performance (23). The administration of LBP to a diet regimen was observed to enhance the growth performance and digestive enzyme activity of broilers (24). Hao et al. demonstrated that the administration of Chinese *wolfberry* and *astragalus* extracts in lieu of a 1% basal diet can enhance the growth performance of Tibetan fragrant pigs (25). The results of this experiment demonstrated that the incorporation of 5% RBWF into the diet led to a notable increase in the dry matter intake of fattening sheep. This was accompanied by a similar trend in both the daily gain and total weight gain. However, no significant effect was observed, which may be attributed to the considerable inter-individual variability within the experimental group. In contrast to the aforementioned findings, Guo et al. (26) observed that the addition of *Lycium barbarum* to the basal diet resulted in a notable enhancement in the average body weight and growth performance of rats. This discrepancy may be attributed to the variation in nutrient digestion and absorption rates resulting from the disparate digestive tract structures of the experimental animals.

Apparent digestibility is a significant indicator of the rate of digestion and absorption of nutrients in the experimental diet. It provides insight into whether the diet meets the nutritional requirements for animal growth (27). The polysaccharide derived from *Lycium barbarum* is a complex mixture of active ingredients with a multitude of potential applications and a promising future in development (28). The study demonstrated that the intake of polysaccharides in the diet exerts a discernible influence on the feed intake and apparent digestibility of nutrients in ruminants (29). Lin et al. (30) demonstrated that LBP enhanced the digestive and absorptive processes and immune function of immunosuppressed mice by inhibiting the activation of the MLCK signaling pathway, and regulated the immune system of the intestinal mucosal barrier. The addition of 5% RBWF resulted in a significantly higher apparent digestibility of OM and NDF compared to the control group. Additionally, there was a notable increase in the apparent digestibility of ADF, from 24.93 to 27.74%. However, this difference was not found to be statistically significant. In contrast to the aforementioned findings, Ju et al. (31) observed that the addition of *Lycium barbarum* polysaccharides to the lamb diet resulted in a notable enhancement in the apparent digestibility of DM, OM, and CP. This discrepancy could be attributed to the distinct processing forms of *Lycium barbarum* products. In this experiment, the fruit was not subjected to extraction, and it was directly incorporated into the lamb diet, consequently influencing the chemical composition of the *Lycium barbarum* additives. In conclusion, the incorporation of RBWF into the diet has been demonstrated to enhance the feed intake of fattening sheep and augment the apparent digestibility of OM and NDF, as well as the daily gain of lambs. This is may achieved by elevating the relative abundance of fiber-degrading bacteria within the rumen, which in turn increases the feed reward.

The compound stomach is the most prominent anatomical feature of ruminants, and the rumen, situated within the four stomachs, serves as the primary site for ruminant digestion of feed. The key indicators for measuring the maintenance of an internal steady state of rumen fermentation in animals are pH, NH_3_-N and VFAs. The pH directly affects the growth and interaction of microorganisms in the rumen, and maintaining an appropriate range is closely related to rumen fermentation. At the same time, the concentration of volatile fatty acids (VFAs) and ammonia nitrogen (NH_3_-N) within the rumen also exert a direct influence on rumen pH (32). NH_3_-N represents the primary nitrogen source for protein synthesis in rumen microorganisms. The microorganisms in the rumen engage in cooperative degradation of nutrients in the feed, converting them into volatile fatty acids (VFAs) that provide energy for body growth (33). The concentration and proportion of VFAs in the rumen are related to dietary composition and energy level, and have an effect on the energy utilization efficiency, growth performance and methane production of the host (34). The addition of 0.6% LBP to the diet of dairy cows has been found to significantly improve the production performance of dairy cows and significantly increase the total volatile fatty acid content of rumen ammonia nitrogen (5). In this study, the concentration of volatile fatty acids (VFAs), including acetate, propionate, iso-butyrate, butyrate and iso-valerate, in rumen fluid demonstrated a linear increase with the elevation of dietary RBWF supplemental level. This outcome aligns with the findings of Duan et al. (35). The pH of rumen fluid in sheep is typically within the range of 5.5–7.0, while the normal range of NH_3_-N is 5.0 to 30 mg/dL (36). The rumen fluid pH and NH_3_-N of all sheep were maintained within the normal range in this experiment. As a consequence of the considerable rise in VFAs concentration in rumen fluid, the pH in the rumen of *Duolang* sheep in each experimental group declined in a linear fashion with the increase of RBWF supplemental level in the diet, while the concentration of NH_3_-N in rumen increased linearly. It may therefore be hypothesized that the addition of RBWF to the diet can increase the protein synthesis of rumen microorganisms, thereby promoting microbial fermentation in the rumen of animals and the absorption and utilization of nutrients in the diet. Further investigation is required to elucidate the precise effects.

The rumen is a digestive organ that contains a diverse range of microorganisms, which work collectively to degrade nutrients in the diet, thereby providing the host with energy (37). Simultaneously, the host provides a stable and conducive environment for the growth of rumen microorganisms, while obtaining energy from the final product (38). The structure and abundance of the rumen microbial flora can exert a direct influence on the health and growth of the host. Conversely, the structure of rumen microbial flora is also influenced by host species, nutritional energy level and environment (39). Prior research has demonstrated that dietary supplementation of *wolfberry* fruit can regulate the composition of gastrointestinal microbiota and cecal fermentation in rabbits (40). The findings of the study conducted indicated that the incorporation of LBP into the weaned piglets diet could potentially enhance the abundance of *Bacteroidetes* in the ileum and cecum, while increasing the levels of *Lactobacillus* and *Bifidobacterium* in the cecum, and the intestinal microflora was shown to be improved (41). Alpha diversity is primarily indicative of the richness and diversity of species distribution within the rumen, whereas beta diversity predominantly reflects the dissimilarities in microbial communities between disparate samples. In this experiment, the rumen microorganisms of *Duolang* sheep were sequenced, and the alpha diversity results demonstrated that the dietary RBWF addition level had no significant impact on the four indexes of Chao1, Ace, Shannon and Simpson in the rumen of *Duolang* sheep. The PCoA and NMDS plots revealed that the bacterial community structures of the different treatment groups were not statistically different from one another, as indicated by the overlap of the respective groups on the plots. The aforementioned results demonstrate that the incorporation of RBWF into the diet does not exert any deleterious effects on the rumen microbial flora structure of *Duolang* sheep.

A substantial body of research has demonstrated that the predominant bacteria in the rumen of ruminants are members of the *Firmicutes* and *Bacteroidota* phyla. These bacteria play a pivotal role in the host’s degradation of complex carbohydrates and promotion of the decomposition and absorption of nutrients (42, 43). The results indicate that *Firmicutes* and *Bacteroidota* play an important role in maintaining the stability of the rumen microbial community. Among them, *Bacteroidota* mainly promotes rumen fermentation to degrade cellulose, soluble sugar and carbohydrates to produce small molecules such as VFAs, which are utilized by the host (44). The role of *Firmicutes* is mainly to enhance host lipid metabolism and promote its energy absorption (45). Fujisaka et al. (46) observed that *Firmicutes* and *Bacteroidota* constituted between 70 and 90% of the total bacterial population. In this experiment, the relative abundance of *Firmicutes* and *Bacteroidota* accounted for approximately 90% of the total bacteria, which was consistent with the aforementioned reports. Furthermore, the relative abundance of *Bacteroidota* and *Fibrobacterota* increased following the addition of RBWF in this experiment, which may facilitate the degradation of cellulose in the rumen and is associated with the enhancement of the apparent digestibility of NDF in this experiment.

In addition to decomposing and metabolizing complex nutrients such as cellulose and protein, *Prevotella* can also engage in cooperative interactions with other microorganisms to facilitate the growth and development of the body (47). The *NK4A214 group* is primarily responsible for the degradation of fibers, which provides energy to the host. Additionally, it has been observed that this process enhances the host’s immunity by inhibiting the growth of harmful bacteria (48). The *Lachnospiraceae NK3A20 group* has also been identified as a potentially beneficial bacterium, with involvement in the metabolism of various carbohydrates and the production of acetic acid and butyric acid, which provide energy for the host (49). *Prevotellaceae UCG 001* primarily degrades proteins and amino acids, producing short-chain fatty acids, and facilitates the degradation of fiber in collaboration with cellulose-decomposing bacteria (50). Following the addition of RBWF to the experimental diet, a notable increase was observed in the relative abundance of *Prevotella*, *NK4A214 group*, *unclassified Prevotellaceae* and *Lachnospiraceae NK3A20 group*. These bacteria are capable of decomposing complex dietary fibers, such as cellulose and pectin, into smaller molecular structures that can be utilized by other bacteria within the rumen, resulting in the production of volatile fatty acids (VFAs). Zhu et al. (51) observed that a reduction in the relative abundance of *Prevotellaceae UCG 001* was associated with an improvement in depressive behavior. This finding was hypothesized to be linked to a decrease in short-chain fatty acids and a reduction in intestinal inflammation. The results of this experiment demonstrated a reduction in the relative abundance of *Prevotellaceae UCG 001*, which may be associated with autoimmune regulation. Furthermore, our study elucidated the correlation between growth performance, nutrient apparent digestibility, rumen fermentation parameters and major bacteria at the genus level.

Prior research has demonstrated a positive correlation between *Prevotellaceae UCG 001* and the expression of inflammatory factors (52). In this experiment, *Prevotellaceae UCG 001* was found to be negatively correlated with feed conversion ratio (FCR) and pH, and positively correlated with other indicators. Furthermore, it was significantly positively correlated with organic matter (OM) apparent digestibility. The concentration of acetic acid and butyric acid in the rumen of *Duolang* sheep in the experimental group increased, which enhanced the absorption of nutrients in the rumen and thus promoted growth performance. The abundance of F082, *Christensenellaceae R7 group*, *NK4A214 group* and *Ruminococcus* was significantly and positively correlated with ADG. An increase in the relative abundance of *NK4A214 group* was observed, resulting in an increase in ADG for the experimental group. However, further study is required to elucidate the specific mechanisms through which these strains affect growth performance and rumen fermentation.

In the context of sheep breeding, economic benefits are closely associated with feed costs and growth performance (53). The results of this experiment indicate that an increase in the proportion of RBWF added to the diet is associated with a reduction in feed unit price. This suggests that the inclusion of RBWF in the diet may offer a potential means of reducing feed costs. However, the total weight gain of sheep in the experimental group with RBWF was found to decrease. In contrast, the H2 group exhibited a 4.78% increase in weight gain compared to the control group. Additionally, the gross profit was observed to increase by 17.45% compared to the control group. These findings suggest that the appropriate amount of RBWF can enhance the profitability of mutton sheep breeding. Furthermore, the 5% addition was identified as the optimal dosage. Han et al. (54) demonstrated that the incorporation of *wolfberry* as a functional or nutritional feed ingredient in broiler diets offers a promising economic benefit. Similarly, Wang et al. (55) revealed that the administration of 0.1–0.2% LBP nutrition lick blocks can markedly enhance the economic viability of fattening Tan sheep. Collectively, these studies highlight the potential of *Lycium barbarum* products to enhance livestock productivity.

## 5 Conclusion

The results of this experiment demonstrate that the administration of RBWF can enhance the growth performance of *Duolang* sheep, optimize rumen fermentation parameters, exert no deleterious effect on the structure and abundance of rumen microbial flora, and augment gross profit. In the context of the experimental conditions, the administration of 5% RBWF yielded more favorable outcomes.

## Data Availability

The datasets presented in this study can be found in online repositories. The names of the repository/repositories and accession number(s) can be found below: https://www.ncbi.nlm.nih.gov/, PRJNA1170910, PRJNA1170932.
