# Supplementary material for: Effects of residual black wolfberry fruit on growth performance, rumen fermentation parameters, microflora and economic benefits of fattening sheep
Source: Front Vet Sci. 2025 Jan 10;11:1528126. doi: 10.3389/fvets.2024.1528126 (PMC11760609; doi:10.3389/fvets.2024.1528126)
Supplement: Supplementary file 1 [file Table_1.DOCX]

Supplementary Material

# Supplementary Tables

**Supplementary Table S1.** Distribution of bacterial taxa averaged under phyla levels across the different treatment groups.

| **Items** | **CK** | **H1** | **H2** | **H3** | **SEM** | ***p*-Value** |
| --- | --- | --- | --- | --- | --- | --- |
| Firmicutes | 54.23 | 44.15 | 54.83 | 54.38 | 3.055 | 0.559 |
| Bacteroidota | 38.47 | 48.72 | 39.70 | 37.09 | 3.271 | 0.574 |
| Proteobacteria | 1.70 | 0.89 | 1.24 | 2.82 | 0.306 | 0.180 |
| Patescibacteria | 1.02 | 1.18 | 1.16 | 0.78 | 0.144 | 0.818 |
| Actinobacteriota | 1.07 | 0.34 | 0.73 | 1.35 | 0.195 | 0.363 |
| Desulfobacterota | 1.40 | 0.61 | 0.32 | 0.50 | 0.201 | 0.178 |
| Fibrobacterota | 0.26 | 2.65 | 0.60 | 0.85 | 0.458 | 0.473 |
| Verrucomicrobiota | 0.51 | 0.56 | 0.57 | 0.51 | 0.071 | 0.991 |
| unclassified_Bacteria | 0.26 | 0.04 | 0.07 | 0.56 | 0.124 | 0.556 |
| Spirochaetota | 0.28 | 0.43 | 0.14 | 0.20 | 0.052 | 0.469 |
| Others | 0.78 | 0.41 | 0.64 | 0.93 | 0.102 | 0.433 |

**Supplementary Table S2.** Distribution of bacterial taxa averaged under genera levels across the different treatment groups.

| **Items** | **CK** | **H1** | **H2** | **H3** | **SEM** | ***p*-Value** |
| --- | --- | --- | --- | --- | --- | --- |
| Prevotella | 12.84 | 20.11 | 17.76 | 16.10 | 1.624 | 0.393 |
| Christensenellaceae_R_7_group | 10.83 | 7.84 | 10.01 | 8.62 | 1.189 | 0.827 |
| NK4A214_group | 5.68 | 5.24 | 10.06 | 8.60 | 1.195 | 0.491 |
| uncultured_rumen_bacterium | 6.50 | 6.92 | 8.03 | 6.22 | 0.670 | 0.843 |
| Ruminococcus | 4.96 | 4.80 | 5.54 | 4.41 | 0.731 | 0.975 |
| Rikenellaceae_RC9_gut_group | 5.97 | 6.43 | 4.92 | 5.53 | 0.863 | 0.974 |
| unclassified_Prevotellaceae | 1.69 | 7.45 | 1.92 | 2.67 | 1.579 | 0.391 |
| Prevotellaceae_UCG_001 | 6.96 | 2.80 | 4.09 | 1.54 | 1.615 | 0.646 |
| Lachnospiraceae_NK3A20_group | 2.70 | 1.19 | 2.78 | 7.66 | 1.364 | 0.469 |
| unclassified_F082 | 1.54 | 2.89 | 1.95 | 1.49 | 0.465 | 0.708 |
| Others | 40.33 | 34.32 | 32.94 | 37.11 | 2.802 | 0.786 |
